# Distinct IgM and IgG autoantibody profiles characterize incomplete and classified systemic autoimmune diseases

**DOI:** 10.3389/fimmu.2026.1847217

**Published:** 2026-06-17

**Authors:** Rebecca A. Wood, Neha Kodali, Nancy Redinger, A. Darise Farris, Christopher J. Lessard, R. Hal Scofield

**Affiliations:** 1 Department of Pathology, University of Oklahoma Health Campus, Oklahoma City, OK, United States; 2 Arthritis & Clinical Immunology, Oklahoma Medical Research Foundation, Oklahoma City, OK, United States; 3 Genes & Human Disease Research Program, Oklahoma Medical Research Foundation, Oklahoma City, OK, United States

**Keywords:** anti-IgG antibody, anti-IgM antibody, autoantibodies, incomplete lupus erythematosus, non-Sjögren’s disease sicca, Sjögren’s Disease (SjD), systemic lupus erythematosus (SLE)

## Abstract

Incomplete lupus erythematosus (ILE) and non-Sjögren’s disease sicca (nSjD-sicca) are clinically heterogeneous, incompletely classified autoimmune conditions that share features with systemic lupus erythematosus (SLE) and Sjögren’s disease (SjD), respectively. Although some patients progress to classified disease, many remain stable. The immunologic features distinguishing incomplete from established autoimmune disease remain poorly defined. We sought to characterize and compare autoantibody immune signatures across ILE, SLE, nSjD-sicca, and SjD using expanded autoantibody profiling. Serum samples were obtained from patients with ILE (n=80), SLE (n=80), nSjD-sicca (n=52), SjD (n=60), and matched healthy controls (n=79). Initial screening was performed using the Bio-Rad BioPlex 2200. Expanded profiling utilized the GeneCopoeia Human Autoimmune Array (120 autoantigens) with parallel IgM and IgG detection. Traditional screening demonstrated expected patterns: ILE (anti-nRNP 26.3%; anti-chromatin 25.0%), SLE (anti-SmRNP 40.0%; anti-dsDNA 31.3%), nSjD-sicca (anti-La 9.6%), and SjD (anti-Ro/SSA 53.3%). Expanded analysis revealed significantly increased IgM autoreactivity in ILE compared with SLE (BH-FDR-adjusted p<0.05), targeting nuclear (KU, Nup62, CENP-A/B), cytokine (IFN-α1, IFN-ϵ, IL-15, GM-CSF), mitochondrial (M2), extracellular matrix (collagen IV, fibrinogen), vascular (β2-glycoprotein I, AGTR), and gut-associated antigens (tissue transglutaminase, intrinsic factor). In contrast, SLE demonstrated enriched IgG responses to canonical nuclear antigens, including core histone and dsDNA. Within the sicca spectrum, Ro52 (TRIM21) IgG autoantibodies were significantly increased in SjD compared to nSjD-sicca. These findings indicate that incomplete autoimmune disease states exhibit distinct IgM-dominant autoantibody profiles rather than simply attenuated versions of the IgG dominant responses in classified disease, highlighting the potential value of isotype-specific profiling for disease classification.

## Introduction

Autoimmune diseases impact about 23.5M US individuals with both autoantibody positivity rates (1) and autoimmune disease prevalence (2) on the rise. Autoimmune disorders occur with loss of immune tolerance to select self-antigens, leading to autoantibody production, inflammation (3) and disease-specific clinical features. Little is known about early etiologic and pathogenic mechanisms of autoimmune diseases. Patients with incomplete phases of autoimmune disease tend to have an initial asymptomatic phase of differing lengths of immune system activation and when autoimmune processes start. After the asymptomatic period, many patients exhibit nonspecific signs and symptoms for years before meeting formal disease classification criteria (3).

Sjögren’s disease (SjD) is a common, systemic autoimmune disease affecting 1M to 4M US invidiuals (4, 5). Those with SjD consist of 90% women with the average age of onset being between 45 to 55 years old (6). Subjects are classified with SjD for research purposes by consensus criteria, such as the 2016 ACR/EULAR criteria (7).Primary SjD is a systemic autoimmune disease with lacrimal and salivary gland dysfunction (4). Nearly all SjD patients develop an increase of autoantibodies circulating their blood stream, with anti-Ro (or SSA) found in 50-70% of patients and anti-La (or SSB) found in about 30% of patients (4). There is currently no cure for SjD available, and treatment options do not fully address the plethora of symptoms of SjD. While some patients with sicca symptoms (dry eyes and/or dry mouth) meet SjD classification, most do not and are classified as non-Sjögren’s Disease-Sicca (nSjD-Sicca). nSjD-Sicca patients typically have lower ANA positivity than SjD, or they have other autoantibodies besides anti-Ro due to it being a part of the 2016 ACR/EULAR SjD classification criteria.

Systemic lupus erythematosus (SLE) is an autoimmune disease that can affect nearly all organ systems, but not the salivary or lacrimal glands. Common symptoms shared by both SLE and SjD include arthritis, fever, vasculitis, interstitial lung disease, neurologic complications, mental fog, and fatigue; however, SLE can also have immune complex glomerulonephritis, serositis, thrombocytopenia, and a malar rash. The 2019 ACR-EULAR classification criteria for SLE includes an antinuclear antibody titer of ≥ to 1:80 with at least one clinical criterion and ≥ 10 points of clinical and immunological criteria (8). Some autoantibodies commonly found within SLE patients include anti-dsDNA (60-70%) (8), anti-Sm (20-40%), anti-nRNP (20-30%) (9), and anti-Ro (30-40%) (10). While there are no cures for SLE, there are several treatment options available including glucocorticoids, hydroxychloroquine, and immunosuppressants, all carrying unpleasant to serious side effects. Like nSjD-Sicca and SjD, many patients suspected of SLE do not meet full disease classification criteria and have been termed incomplete lupus erythematosus (ILE). ILE patients usually have a positive ANA and some clinical or serological feature(s) of SLE but do not meet classification criteria (11).

While each autoimmune disease is unique with different autoimmune reactions and target organs, overlap in genetic associations, environmental risk factors, and pathophysiological mechanisms (12) occur, making the study of two autoimmune diseases, along with their incomplete states, advantageous. Parallel evaluation of nSjD-Sicca and ILE patients will provide unique opportunities to identify common and disease-specific proteomic abnormalities.

Conventional autoantibody testing platforms, such as the BioPlex 2200, assess a limited set of clinically established autoantibody specificities and provide a standardized reference for serologic classification. However, these assays capture only a subset of autoreactive responses and are not specific for each autoimmune disease. Expanded autoantibody arrays enable broader, isotype-resolved profiling across a wider range of antigens, allowing for more comprehensive characterization of immune signatures. Extensive autoantibody studies have been performed in SjD and SLE; however, expanded autoantibody profiling is limited in both nSjD-Sicca and ILE, respectively. The aim of this study is to cross-sectionally examine autoantibody differences between ILE and SLE and between nSjD-Sicca and SjD to determine if there are isotype-resolved serologic profiles that help characterize heterogeneity among incomplete and classified autoimmune disease groups based upon shared or unique autoantibodies.

## Materials and methods

### Participants, clinical characterization and sample processing

This study was approved by the Institutional Review Board of the University of Oklahoma Health Sciences Center and the Oklahoma Medical Research Foundation. Serum samples and clinical data were obtained from the Oklahoma Cohort for Rheumatic Disease (13), Lupus Family Registry and Repository (14), and the Oklahoma Sjögren’s Research Clinic (4). All SLE participants evaluated in this study met the American College of Rheumatology (ACR) SLE classification criteria (ACR ≥4) (15) and all SjD participants met the 2016 American College of Rheumatology/European Alliance of Associations for Rheumatology (ACR/EULAR) criteria for SjD with an objective criteria score ≥4 (15). Demographic information about the participants used in this study are included in **Table 1**. The 1997 ACR SLE classification criteria scores and subcategories are available in **Supplementary Table 1**. The subjective and objective criteria breakdown for the subjects used in this study is in **Supplementary Table 2**, and the lip biopsy interpretations are provided in **Supplementary Table 3**. To be classified as ILE and nSjD-Sicca, they both had symptoms of their disease counterparts, yet they do not meet the classification criteria score to be fully classified as having SLE and SjD, respectively. Individuals were excluded from the study who had been treated with cyclophosphamide or rituximab within the prior 2 years. In addition, no patients treated with calcineurin inhibitors, pulse IV steroids, prednisone > 20mg/day, experimental clinical trial medications or other chemoablative treatments were allowed in the study. A table of certain medications is in **Supplementary Table 4**.

**Table 1:** Participant demographics.

	Controls	ILE	SLE	nSjD-Sicca	SjD
**Total Samples, n**	79	80	80	52	60
Sex, n (%)					
Female	71 (89.7%)	72 (90.0%)	72 (90.0%)	49 (94.2%)	56 (93.3%)
Male	8 (10.3%)	8 (10.0%)	8 (10.0%)	3 (5.8%)	4 (6.7%)
Ancestry Background, n (%)					
White	51 (64.6%)	51 (63.8%)	51 (63.8%)	36 (69.2%)	42 (70.0%)
Black	18 (22.8%)	18 (22.5%)	18 (22.5%)	9 (17.3%)	9 (15.0%)
Asian	5 (6.3%)	6 (7.5%)	6 (7.5%)	3 (5.8%)	4 (6.7%)
Native American	5 (6.3%)	5 (6.2%)	5 (6.2%)	4 (7.7%)	5 (8.3%)
Ethnicity, n (%)					
Non-Hispanic	72 (91.1%)	67 (83.8%)	68 (85%)	48 (92.3%)	52 (86.7%)
Hispanic	7 (8.9%)	13 (16.2%)	12 (15.0%)	4 (7.7%)	8 (13.3%)
Age (Average Years ± Std)	41.7 ± 12.4	42.1 ± 13.2	42.1 ± 12.7	43.5 ± 13.2	46.2 ± 11.8

There were a total of 351 patients in this study. Categorical variables are reported as number (percentage). .

ILE, incomplete lupus erythematosus; SLE, systemic lupus erythematosus; nSjD-Sicca, non Sjögren’s Disease – Sicca; SjD, Sjögren’s Disease.

### Traditional IgG autoantibody testing

Serum autoantibody profiling was performed using the BioPlex 2200 ANA Screen system (Bio-Rad Laboratories, Hercules, CA), a multiplex, bead-based immunoassay that simultaneously detects multiple antinuclear antibodies (ANAs) using fluorescently dyed magnetic beads conjugated to specific autoantigens. The following 12 autoantibodies were measured: Centromere B, Chromatin, double-stranded DNA (dsDNA), La (SS-B), Ribosomal P, RNP (U1-RNP), RNP-A, RNP-68, Ro (SS-A), Scl-70 (topoisomerase I), Sm, and SmRNP. Each sample was processed according to the manufacturer’s instructions. Raw fluorescence intensity was interpreted using Bio-Rad’s proprietary software to generate semi-quantitative antibody index values, with results classified as positive, equivocal, or negative based on predetermined thresholds. All testing was conducted at the Oklahoma Medical Research Foundation (OMRF) Arthritis and Clinical Immunology Phenotyping Core, with internal controls and calibrators included in each run to ensure assay reproducibility and reliability.

### Microarray IgG and IgM autoantibody testing

IgG and IgM autoantibodies were measured using the GeneCopoeia Human Autoimmune Antibody Microarray v2, a high-throughput, glass slide–based protein microarray containing over 120 immobilized autoantigens relevant to systemic autoimmune and inflammatory diseases. The platform enables simultaneous semi-quantitative profiling of autoantibody reactivity against a wide array of nuclear, cytoplasmic, extracellular matrix, and signaling molecules, including antigens associated with systemic lupus erythematosus (SLE), Sjögren’s disease (SjD), systemic sclerosis, myositis, autoimmune hepatitis, and vasculitis.

We followed manufacture’s microarray instructions using their OmicsArray Antigen Microarray Processing Kit (Catalog No. PA100, PA101, PA102, PA103). A list of antigen numbers, antigen IDs, protein name, alternative name/synonyms, gene symbol, and uniprot ID is included in **Supplementary Table 5** (provided by Genecopoeia). Slides were brought to room temperature and washed with 10X PBST. They were then blocked using blocking buffer I for an hour. During this time, serum samples were diluted 1:100 in a DNAse I treatment mixture (75% nuclease free water, 10% 10X DNAse I buffer, 10% 0.1M dithiothreitol, and 5% DNAse I enzyme). After washing, the diluted serum samples were added to the pre-printed antigen arrays at room temperature for 1 hour. After washing, the array was blocked using blocking buffer II. During this time, the secondary antibody mixture (1:1000 dilutions of Cy3 anti-human IgG or Cy5 anti-human IgM with 1X PBST) was made. After incubation and washing with 1X PBS, slides were scanned using a GenePix 4400B microarray scanner at standardized PMT settings.

Signal intensities were extracted using GenePix Pro software. Raw array data were analyzed using previously developed pipelines (16, 17). Raw data were extracted as median fluorescence intensity values. Replicate spot intensities were averaged per autoantibody, and PBS control intensities were subtracted to generate background-corrected signal. Negative values were set to zero to avoid non-physical fluorescence measurements. Background-corrected signals were then adjusted using signal-to-noise ratio (SNR) factors. Adjusted signals were subsequently log2-transformed (log2(signal + 1)) to reduce right-skewness and stabilize variance across features.

Autoantibodies were considered positive if their signal intensity exceeded the mean + 2 standard deviations of healthy control values for each antigen. To evaluate the impact of positivity threshold selection, we performed a sensitivity analysis using alternative cutoff strategies based on control samples. In addition to the primary thresholding approach, we applied (1) a parametric cutoff defined as the mean plus two standard deviations (mean + 2SD), (2) a slightly less stringent parametric cutoff (mean + 1.8SD), and (3) a non-parametric cutoff defined as the 95th percentile of control values. Positivity rates were calculated for each method and compared across groups to assess the stability. Observed slides included positive and negative controls for quality control, and antigens were printed in duplicates. Arrays failing internal control QC metrics (e.g., low signal-to-noise ratio, uneven hybridization) were excluded.

### Data analysis and statistical methods

All statistical analyses were performed using R version 4.5.1 and GraphPad Prism version 10.6.1. BioPlex 2200 data were analyzed both as semi-quantitative index values and dichotomized as positive/negative per manufacturer’s cutoffs. Autoantibody signal intensities were analyzed using non-parametric statistical tests, given the non-Gaussian distribution of the data. Between-group comparisons (e.g., ILE vs SLE, SjD vs nSjD-Sicca) were performed using the Mann–Whitney U test for two-group comparisons or the Kruskal–Wallis test followed by Dunn’s *post hoc* test for three or more groups.

Differential autoantibody reactivity was assessed using linear modeling implemented in the *limma* package. Analyses were performed separately for IgG and IgM datasets using log2-transformed continuous signal intensities. Group comparisons were performed using *limma* with Benjamini-Hochberg correction. These analyses constituted the primary statistical framework for identifying differentially reactive autoantibodies. For each comparison (e.g., ILE vs SLE, control vs SjD, control vs nSjD-sicca), a design matrix was constructed to model group membership as the primary variable. Linear models were fit f or each autoantibody feature, and empirical Bayes moderation was applied to improve variance estimation across features. Pairwise contrasts between groups were defined within the model framework, and moderated t-statistics were used to identify differentially reactive autoantibodies. Resulting p-values were adjusted for multiple testing using the Benjamini–Hochberg false discovery rate (BH-FDR) method. Autoantibodies with BH-FDR-adjusted p-values ≤ 0.05 were considered statistically significant.

Heatmaps, scatterplots, and volcano plots were used to visualize autoantibody-specific reactivity patterns Volcano plots were generated using the ggplot2 package in R and were used to visualize differential IgG or IgM reactivity between groups, plotting log_2_ fold change versus –log_10_(BH-FDR-adjusted p-values). Prism was used for heat maps and grouped scatterplots. Missing data were excluded pairwise, and no imputation was performed.

### Positivity-based analyses

Positivity thresholds were applied as a secondary descriptive approach to summarize the prevalence and distribution of autoantibody reactivity across groups. Positivity-based analyses were not used as the primary method for identifying differentially reactive autoantibodies.

## Results

### Traditional autoantibody screening reveals distinct serologic profiles across clinical groups

Initial IgG autoantibody screening using a multiplex panel assessing twelve common autoantibody specificities demonstrated distinct serologic patterns across clinical groups. Among ILE subjects, the most frequent reactivities were anti-nRNP (26.3%) and anti-chromatin (25.0%), whereas SLE patients exhibited higher frequencies of anti-SmRNP (40.0%) and anti-dsDNA (31.3%). In the sicca spectrum, nSjD-sicca showed low-level autoreactivity, most commonly anti-La (9.6%) and anti-centromere B (5.8%), while Sjögren’s disease (SjD) was characterized by prominent anti-Ro (53.3%) and anti-La (26.7%) positivity (**Table 2**). These findings are consistent with established disease-associated autoantibody profiles and provide a framework and validation reference for expanded isotype-specific analysis.

**Table 2:** Frequency of traditional autoantibody specificities across clinical groups.

Autoantibody positivity, n (%)	ILE	SLE	nSjD-Sicca	SjD
Total, n	80	80	52	60
Centromere B	5 (6.3%)	3 (3.8%)	3 (5.8%)	9 (15.0%)
Chromatin	20 (25%)	35 (43.8%)	0 (0.0%)	5 (8.3%)
dsDNA	11 (13.8%)	25 (31.3%)	0 (0.0%)	0 (0.0%)
La/SSB	3 (3.8%)	5 (6.3%)	5 (9.6%)	16 (26.7%)
Ribosomal P	3 (3.8%)	6 (7.5%)	0 (0.0%)	1 (1.7%)
RNP	21 (26.3%)	29 (36.3%)	4 (7.7%)	6 (10.0%)
RNP-A	21 (26.3%)	27 (33.8%)	N/A	N/A
RNP_68	4 (5.0%)	3 (3.8%)	N/A	N/A
Ro/SSA	8 (10.0%)	19 (23.8%)	3 (5.8%)	32 (53.3%)
Scl-70	2 (3.0%)	1 (1.3%)	0 (0.0%)	1 (1.7%)
Sm	7 (8.8%)	19 (23.8%)	0 (0.0%)	2 (3.3%)
SmRNP	15 (18.8%)	32 (40.0%)	0 (0.0%)	3 (5.0%)

Autoantibody positivity is shown as number of positive subjects and percentage within each disease group. Serum samples from patients with incomplete lupus erythematosus (ILE; n=80), systemic lupus erythematosus (SLE; n=80), non-Sjögren’s disease sicca (nSjD-sicca; n=52), and Sjögren’s disease (SjD; n=60) were analyzed using the bio-rad bioplex 2200 multiplex bead-based assay according to the manufacturer’s protocol. Positivity was defined based on instrument-specific cutoff thresholds. RNP-A and RNP_68 were not assessed in the nSjD-sicca and SjD cohorts (N/A). Percentages are calculated within each disease group.

### Expanded autoantibody profiling reveals broad IgM autoreactivity in ILE compared to SLE

Expanded autoantibody array testing using the GeneCopoeia Human Autoimmune Microarray v2 showed that IgM and IgG autoantibody signals demonstrated substantial heterogeneity across individual samples, particularly for IgM. Despite this variability, group-level differences were identified through differential analysis. Full differential analysis results are in **Supplementary Table 8**.

**Figure 1:**
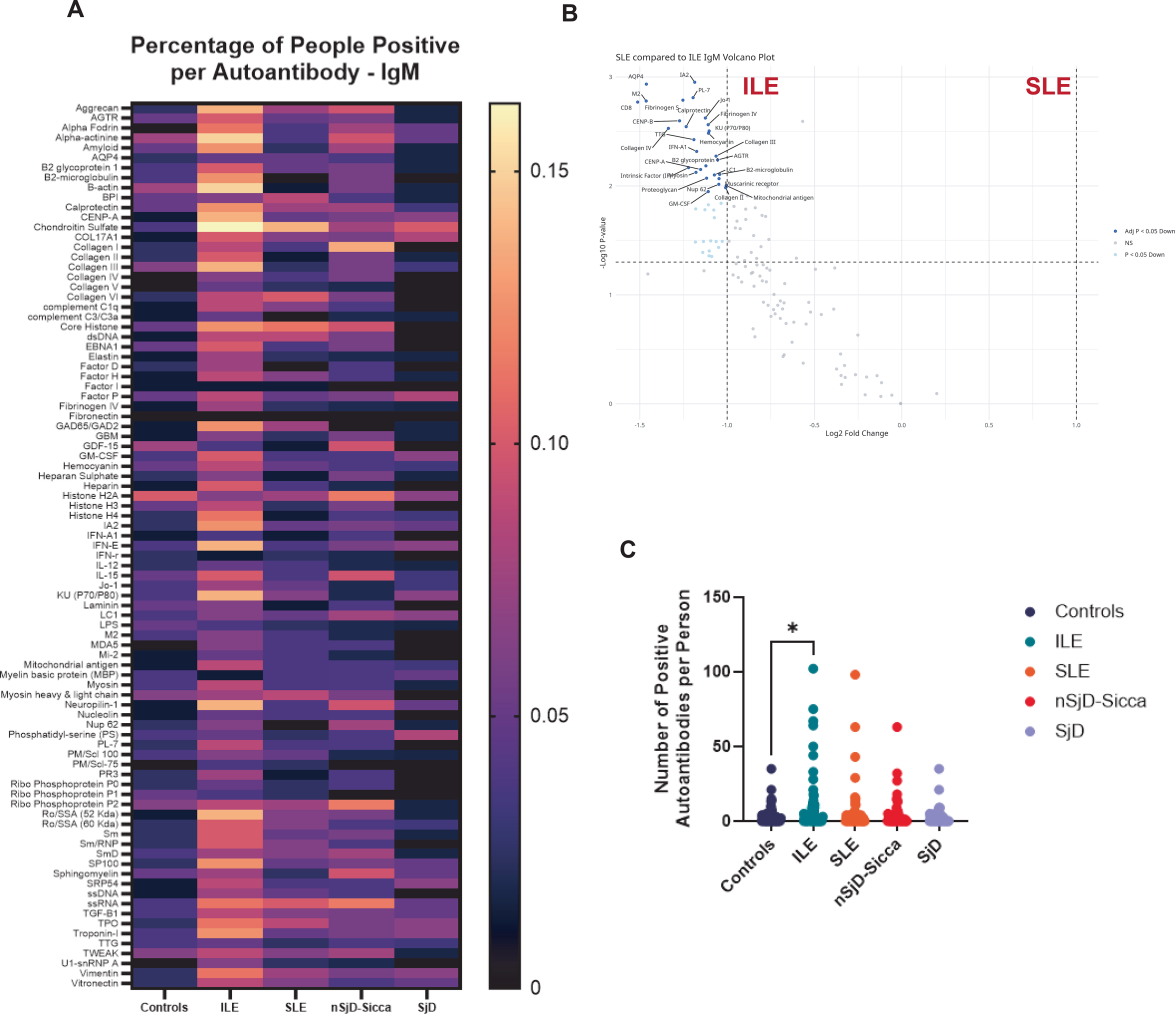
IgM autoantibody profiles distinguish incomplete and classified systemic autoimmune disease states. **(A)** Heatmap showing the percentage of individuals positive for each IgM autoantibody across cohort-matched healthy controls, ILE, SLE, nSjD-sicca, and SjD. Positivity was defined as two standard deviations above healthy control means, and color intensity reflects the proportion of subjects positive per autospecificity. Any autoantibodies that had low percentage of people positive per autoantibody (<10%) for each group were removed from the figure. **(B)** Volcano plot comparing IgM autoantibody reactivity between ILE and SLE using limma-based differential analysis with false discovery rate (BH-FDR) adjustment. Log_2_ fold change is shown on the x-axis and −log_10_ adjusted p-values on the y-axis. Autoantibodies with significantly increased IgM reactivity in ILE or SLE after BH-FDR correction are highlighted. **(C)** Number of IgM autoantibodies positive per individual across study groups. Each point represents a single subject, with group distributions shown. ILE demonstrates a higher burden of IgM autoreactivity compared with SLE. Kruskal Wallis with Dunn’s multiple comparisons test was performed using q ≤ 0.05 as significant. ILE, incomplete lupus erythematosus; SLE, systemic lupus erythematosus; nSjD-Sicca, non Sjögren’s Disease – Sicca; SjD, Sjögren’s Disease.

IgM autoantibody reactivity in ILE compared to SLE after correction for multiple testing was significantly increased (BH-FDR-adjusted p < 0.05; **Figures 1A, B**). When comparing nSjD-Sicca to SjD, there was a slight increase in the percentage of individuals positive for IgM autoantibodies in nSjD-Sicca compared to SjD, however, it was not statistically significant (**Figures 1A, C**). IgM autoantibodies targeting nuclear and chromatin-associated antigens were elevated in ILE, including CENP-A (adj. p = 0.042), CENP-B (adj. p = 0.030), Ku (P70/P80) (adj. p = 0.030), and Nup62 (adj. p = 0.044).

In addition, IgM responses directed against cytokine and immune signaling targets were increased in ILE compared to SLE, including IFN-α1 (adj. p = 0.039), IFN-ϵ (adj. p = 0.048), IL-15 (adj. p = 0.048), and GM-CSF (adj. p = 0.047) (**Figure 1B**). These reactivities may suggest heightened early autoreactive responses or regulatory feedback in incomplete disease stages.

Broader IgM autoreactivity in ILE also extended to mitochondrial and organelle-associated antigens, such as M2 (adj. p = 0.030), as well as extracellular matrix and structural proteins, including collagen IV (adj. p = 0.030) and fibrinogen S (adj. p = 0.030). Additional IgM targets included vascular and coagulation-related antigens, such as β2-glycoprotein I (adj. p = 0.042) and angiotensin II receptor type 1 (AGTR) (adj. p = 0.041), along with gut-associated antigens, including tissue transglutaminase (adj. p = 0.032) and intrinsic factor (adj. p = 0.042). Collectively, these findings demonstrate a broad and diverse IgM autoreactive signature in ILE, consistent with patterns that may reflect differences in isotype class switching across autoimmune states (**Figure 1B**; **Supplementary Table 3**). When looking at the number of autoantibodies positive per person in each group, ILE had a statistically significant increase in the number of IgM-specific autoantibodies per person compared to healthy controls (**Figure 1C**). Comparisons of ILE and controls volcano plots for IgM and IgG are shown in **Supplementary Figure 1**.

### SLE Is characterized by canonical IgG autoantibody responses

**Figure 2:**
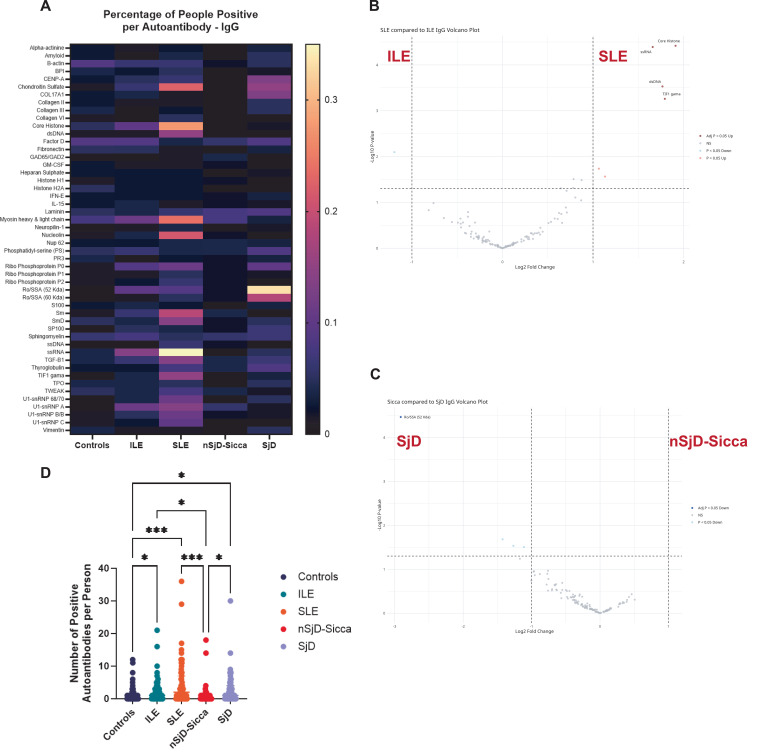
IgG autoantibody profiles reflect isotype specificity in SLE and SjD. **(A)** Heatmap showing the percentage of individuals positive for each IgG autoantibody across cohort-matched healthy controls, incomplete lupus erythematosus (ILE), systemic lupus erythematosus (SLE), non-Sjögren’s disease sicca (nSjD-sicca), and Sjögren’s disease (SjD). Positivity was defined as two standard deviations above control means, and color intensity reflects the proportion of subjects positive per autoantibody. Any autoantibodies that had low percentage of people positive per autoantibody (<10%) for each group were removed from the figure. **(B)** Volcano plot comparing IgG autoantibody reactivity between SLE and ILE using limma-based differential analysis with false discovery rate (BH-FDR) adjustment. Log_2_ fold change is shown on the x-axis and −log_10_ adjusted p-values on the y-axis. Canonical nuclear and chromatin-associated IgG autoantibodies significantly increased in SLE after BH-FDR correction are highlighted. **(C)** Volcano plot comparing IgG autoantibody reactivity between SjD and nSjD-sicca using limma-based analysis with BH-FDR adjustment. Anti-Ro52 (TRIM21) IgG demonstrates significantly increased reactivity in SjD compared with nSjD-Sicca. **(D)** Scatterplot showing the number of positive autoantibodies positive per person. Kruskal Wallis with Dunn’s multiple comparisons test was performed using q ≤ 0.05 (*), q ≤ 0.01 (**), and q ≤ 0.001 (***). SLE and SjD exhibit increased IgG autoantibody burden compared with incomplete or non-classified disease states.

In contrast to the IgM-dominant profile observed in ILE, IgG autoantibody reactivity was significantly more frequent with broader targets in SLE. SLE sera demonstrated increased IgG targeting core histone (adj. p = 0.0026), double-stranded DNA (adj. p = 0.0126), and single-stranded RNA (adj. p = 0.0026) compared with ILE (**Figure 2B**). These reactivities correspond to established SLE-associated targets and are potentially consistent with class-switched, nucleic acid–directed autoimmunity. IgG autoantibodies were also detected against nuclear regulatory proteins in SLE, including TIF1-γ (adj. p = 0.0176) (**Figure 2B**). **Supplementary Figure 2** shows differential autoantibody reactivity in SLE compared to controls.

### Anti-Ro52 IgG distinguishes Sjögren’s disease from non-Sjögren’s sicca

In contrast to the broader patterns observed in the lupus spectrum, the sicca-spectrum comparison yielded more limited differences, with anti-Ro52 (TRIM21) IgG representing the most consistent finding after correction for multiple testing with a significant increase in anti-Ro52 (TRIM21) IgG in Sjögren’s disease compared with nSjD-sicca (adj. p = 0.0044) (**Figure 2C**). No additional IgG or IgM autoantibody specificities differed significantly between SjD and nSjD-sicca after BH-FDR correction, although anti-Ro60, U1-snRNP 68/70, and RM/Scl 100 IgG autoantibodies trended higher with differences noted before correction for multiple testing. The number of IgG autoantibodies present in SjD was statistically elevated, indicating a traditional autoantibody pattern of increased anti-Ro and anti-La compared to nSjD-Sicca (**Figure 2D**). **Supplementary Figures 3** and **4** show differential autoantibody reactivity in nSjD-Sicca compared to controls and SjD compared to controls, respectively.

### Summary of key serologic differences

Together, these results show that ILE and nSjD-sicca are characterized by limited or absent IgG autoreactivity and broader IgM-dominant profiles, whereas SLE and SjD exhibit more antigenically focused, IgG-dominant responses targeting canonical disease-associated autoantigens. These distinct isotype-specific patterns highlight differences in serologic profiles across autoimmune disease states and support the use of isotype-resolved profiling to better characterize disease heterogeneity. Full heatmaps of all 120 autoantibodies and disease groups are found in **Supplementary Figure 5** for IgM and **Supplementary Figure 6** for IgG.

## Discussion

This study demonstrates that IgM and IgG autoantibody profiles diverge across autoimmune disease states, revealing distinct serologic profiles in both the ILE/SLE and nSjD-Sicca/Sjögren’s disease spectra. By leveraging high-dimensional autoantigen arrays and clinical ANA profiling, we identify isotype-specific signatures that distinguish early or incomplete autoimmune phenotypes from classified systemic autoimmune diseases, with differences in IgM- and IgG-dominant reactivity. This study used a discovery-oriented autoantigen array in selected autoimmune cohorts, low positivity rates for individual traditional autoimmune disease associated antigens should not be interpreted as evidence against their clinical utility in diagnostic testing panels since the Bioplex2200 is not specific for ILE, SLE, nSjD-Sicca, and SLE screening.

Continuous signal-intensity analyses served as the primary framework for identifying differentially reactive autoantibodies, whereas positivity-based analyses provided complementary descriptive information regarding the distribution of autoreactivity across disease groups.

In the lupus spectrum, ILE patients displayed broad IgM autoreactivity across a range of nuclear, structural, cytokine, and tissue-specific antigens. This IgM pattern was not restricted to classic lupus targets, but included diverse antigens involved in interferon signaling (e.g., IFN-α1, IFN-ϵ, IL-15), apoptotic debris clearance (e.g., mitochondrial antigens, CD8, calprotectin), and mucosal and barrier integrity (e.g., tissue transglutaminase, intrinsic factor). Many of these antigens are recognized by natural or polyreactive IgM clones involved in homeostatic clearance, and their increased reactivity in ILE compared to SLE is consistent with relatively greater IgM-dominant reactivity.

The presence of IgM reactivity in ILE aligns with recent studies showing that transitional and marginal zone-like B cells populations in incomplete or non-classified autoimmune states, which are often associated with low-affinity IgM autoreactivity. These findings are consistent with models proposing that broadened IgM autoreactivity may be linked to differences in B cell activation and selection processes, whereas IgG-dominant responses in SLE have been associated with antigen-specific refinement and T cell-dependent germinal center activity.

In contrast, SLE patients exhibited antigen-focused IgG responses to established chromatin antigens—such as dsDNA, histones, and ribonucleoproteins—as well as reactivity to nuclear regulatory proteins like TIF1-γ. These IgG specificities are consistent with class-switched, affinity-matured B cell responses driven by nucleic acid-sensing pathways, particularly TLR7 and TLR9 activation in plasmacytoid dendritic cells and autoreactive B cells; however, these possibilities were not directly examined in the present study. The enrichment of IgG in SLE, compared to the IgM-dominant signature in ILE, highlights distinct isotype-resolved immune profiles, with SLE characterized by IgG-dominant autoreactivity that aligns with established features of antigen-specific, T cell-dependent humoral responses.

Within the sicca spectrum, Ro52 (TRIM21) IgG emerged as the most discriminatory autoantibody between SjD and nSjD-Sicca, consistent with its vital role in Sjögren’s classification and pathogenesis. Ro52 is an interferon-inducible E3 ligase highly expressed in epithelial cells during stress and apoptosis. Its chronic exposure in salivary and lacrimal glands drives antigen presentation and germinal center formation, resulting in high-affinity IgG responses. Importantly, anti-Ro52 IgG was elevated across all objective SjD diagnostic criteria, including lip biopsy, ocular staining, Schirmer’s testing, and salivary flow, reinforcing its clinical and biological relevance.

In contrast, nSjD-Sicca subjects demonstrated minimal IgG autoreactivity, even among those meeting single clinical criteria. The absence of Ro52 IgG in this group could suggest that subjective sicca symptoms may arise independently of systemic autoimmunity or reflect local inflammation without differences in B-cell responses; further studies will need to be done to determine the underlying mechanism. Notably, nSjD-sicca patients exhibited mild IgM reactivity in some cases, which could reflect low-level or heterogeneous humoral immune response.

Together, these findings support a model in which autoimmune disease states differ along isotype and antigen-specific dimensions. IgM-dominant autoreactivity is more prominent in incomplete or non-classified disease groups, whereas classified diseases such as SLE and SjD exhibit more focused, IgG-dominant responses. While these cross-sectional data do not establish temporal progression, they highlight distinct isotype-resolved profiles across both lupus and sicca-spectrum phenotypes.

This study has several limitations. First, the cross-sectional design prevents tracking individual immune progression from IgM to IgG. Longitudinal studies are needed to determine whether IgM reactivity predicts future IgG seroconversion or clinical transition. Second, while the array platform provides broad coverage, not all antigens have been functionally validated, and the clinical relevance of some IgM targets remains unclear. Third, the study cohorts were moderately sized, and findings should be validated in additional independent populations with prospective follow-up. Lastly, Although medication use and exclusion criteria were summarized and reported, the present study was not designed or powered to formally adjust for treatment exposure, disease activity, disease duration, or organ involvement. Therefore, the potential influence of these clinical factors on autoantibody profiles cannot be excluded. Future studies with larger cohorts and more detailed clinical covariate data will be necessary to determine the extent to which treatment exposure contributes to the observed serologic differences.

This study demonstrates that autoantibody isotype and target specificity can distinguish incomplete and classified autoimmune phenotypes. ILE and nSjD-Sicca patients exhibited broad IgM autoreactivity with limited IgG responses. In contrast, SLE and Sjögren’s disease were characterized by focused IgG responses, IgG-dominant reactivity against canonical nuclear antigens. These findings define distinct isotype-resolved serologic profiles across disease groups and highlight the potential utility of isotype-specific profiling for improving the characterization of autoimmune disease heterogeneity.

## Data Availability

The raw data supporting the conclusions of this article will be made available by the authors, without undue reservation.
