# Supplementary material for: Distinct IgM and IgG autoantibody profiles characterize incomplete and classified systemic autoimmune diseases
Source: Front Immunol. 2026 Jun 17;17:1847217. doi: 10.3389/fimmu.2026.1847217 (PMC13318661; doi:10.3389/fimmu.2026.1847217)
Supplement: Supplementary file 1 [file SupplementaryFile1.docx]

|  | ***ILE**  **(n=77)** | **SLE**  **(n=80)** |
| --- | --- | --- |
|  |  |  |
| **Score (Average ± SD)** | 2.9 [SD=0.9] | 6.0 [SD=1.7] |
|  |  |  |
| **ACR 1997 Criteria Subcategories (n, %)** | | |
|  |  |  |
| **Malar Rash** | 11 (14.3%) | 45 (56.3%) |
| **Discoid Rash** | 8 (10.4%) | 18 (22.5%) |
| **Photosensitivity** | 16 (20.8%) | 41 (51.3%) |
| **Oral Ulcers** | 13 (16.9%) | 41 (51.3%) |
| **Arthritis** | 44 (57.1%) | 69 (86.3%) |
| **Serositis Combined** | 0 (0.0%) | 29 (36.3%) |
| ***Pleuritis** | 0 (0.0%) | 25 (31.3%) |
| ***Pericarditis** | 0 (0.0%) | 10 (12.5%) |
| **Renal Combined** | 3 (3.9%) | 29 (36.3%) |
| ***Proteinuria** | 3 (4.4%) | 29 (36.3%) |
| ***Cellular Casts** | 0 (0.0%) | 4 (5.0%) |
| **Neurologic Combined** | 2 (2.6%) | 11 (13.8%) |
| ***Seizures** | 1 (1.5%) | 7 (8.8%) |
| ***Psychosis** | 0 (0.0%) | 5 (6.3%) |
| **Hematologic Combined** | 20 (26.0%) | 48 (60.0%) |
| ***Hemolytic Anemia** | 1 (1.5%) | 5 (6.3%) |
| ***Leukopenia** | 12 (17.6%) | 34 (42.5%) |
| ***Lymphopenia** | 11 (16.2%) | 32 (40.0%) |
| ***Thrombocytopenia** | 1 (1.5%) | 15 (18.8%) |
| **Immunologic Combined** | 33 (42.9%) | 68 (85.0%) |
| ***Anti-dsDNA** | 18 (26.5%) | 52 (65.0%) |
| ***Anti Sm** | 3 (4.4%) | 25 (31.3%) |
| ***Anti CL** | 23 (33.8%) | 46 (57.5%) |
| ***Anti-lupus coagulant** | 6 (8.8%) | 8 (10.0%) |
| ***FalsePosVDRL** | 2 (2.9%) | 5 (6.3%) |
| ***LECell** | 0 (0.0%) | 1 (1.3%) |
| **ANA** | 77 (100.0%) | 80 (100.0%) |
|  |  |  |

**Supplementary Table 1.** American College of Rheumatology (ACR) 1997 classification criteria subcategories represented in the incomplete lupus erythematosus (ILE) and systemic lupus erythematosus (SLE) cohorts as outlined in (3). Clinical and immunologic manifestations were categorized according to the revised 1997 ACR classification criteria for SLE. Counts and percentages of each criterion are shown for each cohort. Criteria were assessed through clinical evaluation, medical record review, and serologic testing. *ILE had three subjects with missing ACR 1997 criteria and missing subcategory information for nine subjects.

|  |  |
| --- | --- |

|  | **nSjD-Sicca (n=52)** | **SjD**  **(n=60)** |
| --- | --- | --- |
|  |  |  |
| **Subjective Criteria, n (%)** |  |  |
|  |  |  |
| Dry Eyes | 47 (90.4%) | 57 (95.0%) |
| Dry Mouth | 49 (94.2%) | 57 (95.0%) |
|  |  |  |
| **Objective Criteria, n (%)** |  |  |
|  |  |  |
| Anti-Ro/SSA Positive | 3 (5.8%) | 32 (53.3%) |
| Ocular Staining Score | 14 (26.9%) | 27 (45.0%) |
| Schirmers Test | 11 (21.2%) | 29 (48.3%) |
| Whole Unstimulated Salivary Flow | 18 (34.6%) | 40 (66.7%) |
| Focus score ≥ 1 foci/4 mm^2^ | 8 (15.4%) | 47 (78.3%) |

**Supplementary Table 2**. Subjective and objective criteria of nSjD-Sicca and SjD samples according to the 2016 ACR/EULAR criteria as outlined in Reference 19.

nSjD-Sicca, non Sjögren’s Disease but with signs/symptoms of dryness (Sicca);

SjD, Sjögren’s Disease.

|  |  |
| --- | --- |

|  | nSjD-Sicca (n=52) | SjD  (n=60) |
| --- | --- | --- |
|  |  |  |
| Lip Biopsy Interpretation, n (%) | | |
|  |  |  |
| Non-specific chronic inflammation | **33 (63.5%)** | **7 (11.7%)** |
| Focal lymphocytic sialadenitis (salivary inflammation) | **11 (21.2%)** | **39 (65.0%)** |
| Chronic sialadenitis | **0 (0%)** | **1 (1.7%)** |
| Normal | **3 (5.8%)** | **1 (1.7%)** |
| Unknown | **4 (7.7%)** | **9 (15.0%)** |

**Supplementary Table 3.** Lip Biopsy Interpretation for nSjD-Sicca and SjD subjects.

nSjD-Sicca, non Sjögren’s Disease – Sicca; SjD, Sjögren’s Disease.

|  |  |
| --- | --- |

|  | **Controls (n=79)** | **ILE**  **(n=77)** | **SLE**  **(n=80)** | **nSjD-Sicca (n=52)** | **SjD**  **(n=60)** |
| --- | --- | --- | --- | --- | --- |
| **Medications, n (%)** | |  |  |  |  |
|  |  |  |  |  |  |
| Hydroxychloroquine | 3 (3.8%) | 43 (55.8%) | 67 (83.8%) | 6 (11.5%) | 19 (31.7%) |
| Quinacrine | 0 (0.0%) | 0 (0.0%) | 4 (5.0%) | 0 (0.0%) | 0 (0.0%) |
| Sulfasalazine | 0 (0.0%) | 0 (0.0%) | 0 (0.0%) | 0 (0.0%) | 1 (1.7%) |
| Methotrexate | 0 (0.0%) | 3 (3.9%) | 10 (12.5%) | 1 (1.9%) | 5 (8.3%) |
| Azathioprine | 0 (0.0%) | 0 (0.0%) | 14 (17.5%) | 2 (3.8%) | 0 (0.0%) |
| Mycophenolate Mofetil | 0 (0.0%) | 1 (1.3%) | 17 (21.3%) | 2 (3.8%) | 1 (1.7%) |
| Prednisone < 30 | 0 (0.0%) | 14 (18.2%) | 22 (27.5%) | 6 (11.5%) | 2 (3.4%) |
| Prednisone > 30 | 0 (0.0%) | 4 (5.2%) | 22 (27.5%) | 0 (0.0%) | 0 (0.0%) |
| Belimumab | 0 (0.0%) | 0 (0.0%) | 5 (6.3%) | 0 (0.0%) | 0 (0.0%) |
| Etanercept | 0 (0.0%) | 0 (0.0%) | 1 (1.3%) | 1 (1.9%) | 0 (0.0%) |
| Abatacept | 0 (0.0%) | 0 (0.0%) | 1 (1.3%) | 0 (0.0%) | 0 (0.0%) |
| Cevimeline | 0 (0.0%) | 0 (0.0%) | 0 (0.0%) | 5 (9.6%) | 6 (10.0%) |
| Pilocarpine | 0 (0.0%) | 0 (0.0%) | 0 (0.0%) | 0 (0.0%) | 2 (3.3%) |

**Supplementary Table 4.** Medication usage among study participants. Current and/or prior medications used by participants in each cohort are shown, including immunomodulatory, immunosuppressive, corticosteroid, and symptomatic therapies where applicable. Data are presented as the number and percentage of participants receiving each medication at the time of sample collection or clinical assessment. Individuals were excluded from the study who had been treated with cyclophosphamide or rituximab within the prior 2 years. In addition, no patients treated with calcineurin inhibitors, pulse IV steroids, prednisone > 20mg/day, experimental clinical trial medications or other chemoablative treatments were allowed in the study.

|  |  |
| --- | --- |

| **Method:** | **Percent of Positive Controls** |
| --- | --- |
| Mean + 2SD | 0.025414 |
| Mean + 1.8SD | 0.051581 |
| 95th Percentile | 0.051581 |

**Supplementary Table 6**. **Comparison of positivity threshold strategies based on control samples.**Average control positivity rates were calculated using three thresholding approaches: mean + 2 standard deviations (SD), mean + 1.8 SD, and the 95th percentile of control values. The mean + 2SD approach yielded a lower-than-expected positivity rate (2.54%), indicating a relatively stringent cutoff. In contrast, both the mean + 1.8SD and 95th percentile approaches produced positivity rates of ~5.16%, consistent with the expected false positive rate. These results demonstrate the impact of threshold selection on positivity frequency.

|  |  |
| --- | --- |

**
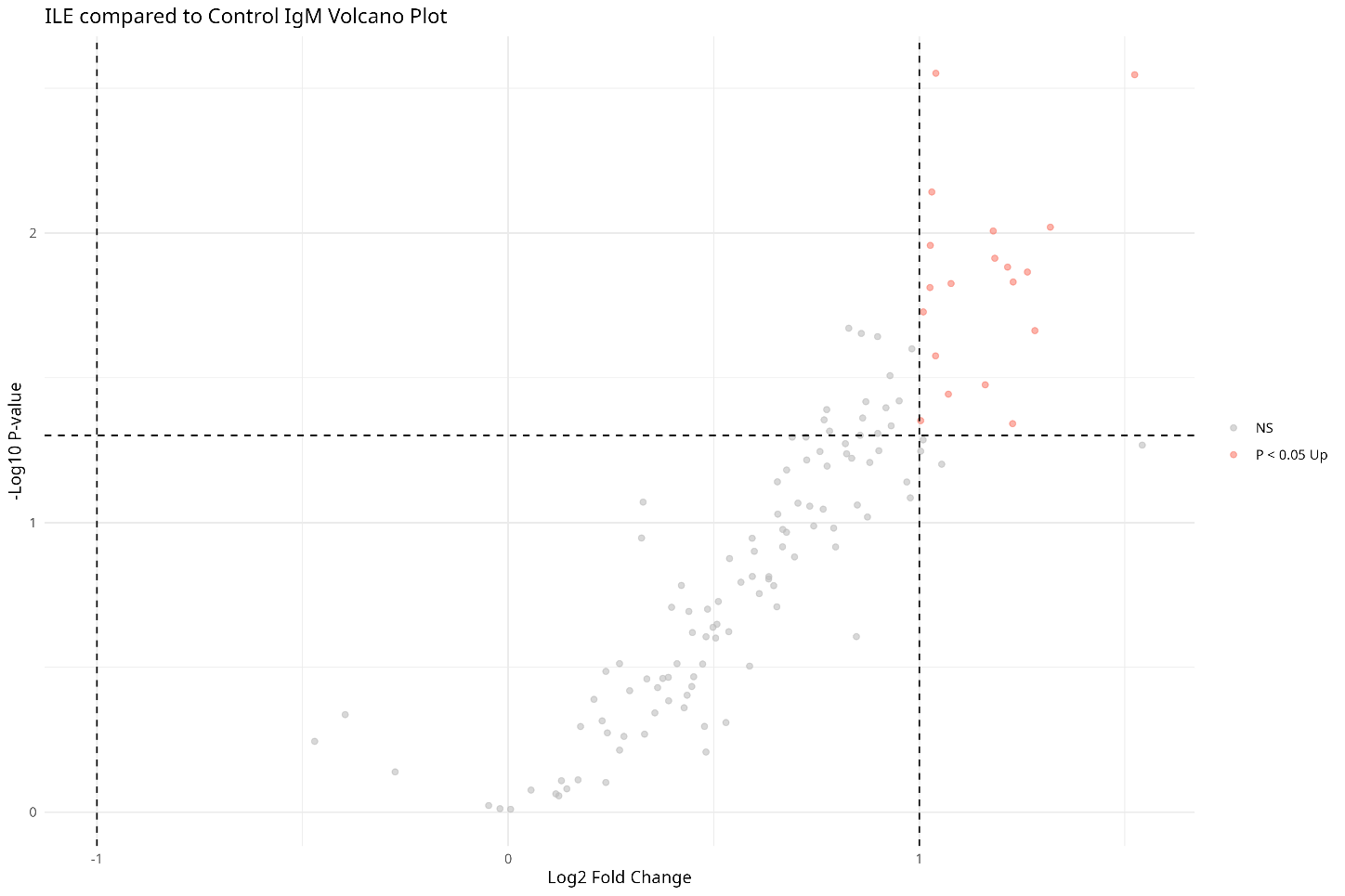
** **
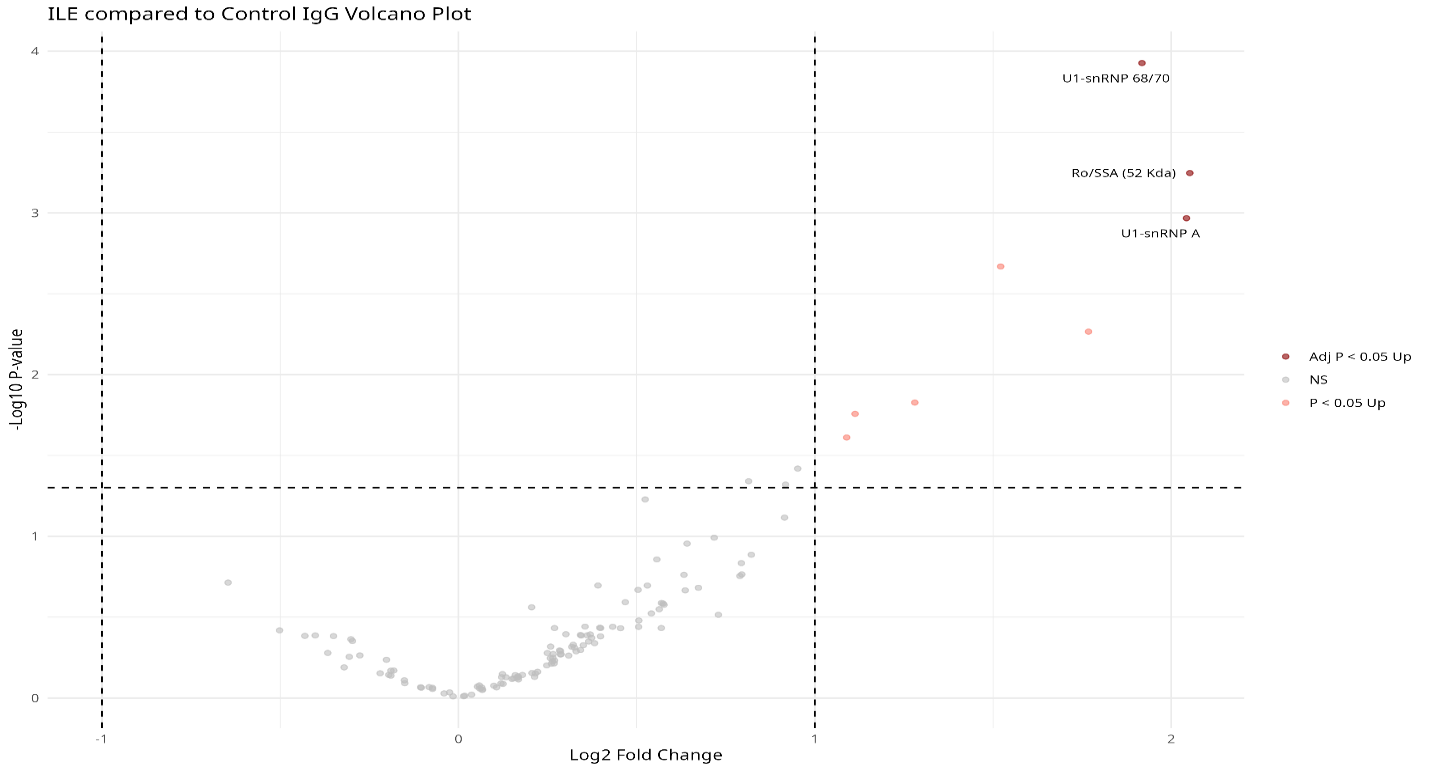
**

**Supplementary Figure 1. Differential autoantibody reactivity in incomplete lupus erythematosus (ILE) compared to healthy controls.**(A) Volcano plot demonstrating differential IgM autoantibody reactivity between ILE and healthy control sera measured using the GeneCopoeia Human Autoimmune Profiling Array. The x-axis represents log2 fold change and the y-axis represents –log10 adjusted p value. Vertical dashed lines indicate fold change thresholds and the horizontal dashed line indicates the significance threshold (adjusted p < 0.05). Red points represent significantly elevated autoantibodies in ILE compared to controls, while gray points indicate non-significant autoantibodies. (B) Volcano plot demonstrating differential IgG autoantibody reactivity between ILE and healthy controls. Significant autoantibodies are highlighted in red and labeled where applicable, including U1-snRNP 68/70, Ro/SSA (52 kDa), and U1-snRNP A. The x-axis represents log2 fold change and the y-axis represents –log10 adjusted p value. Statistical analyses were performed using limma with BH-FDR multiple comparison correction.

|  |  |
| --- | --- |

**B**

**A**

**
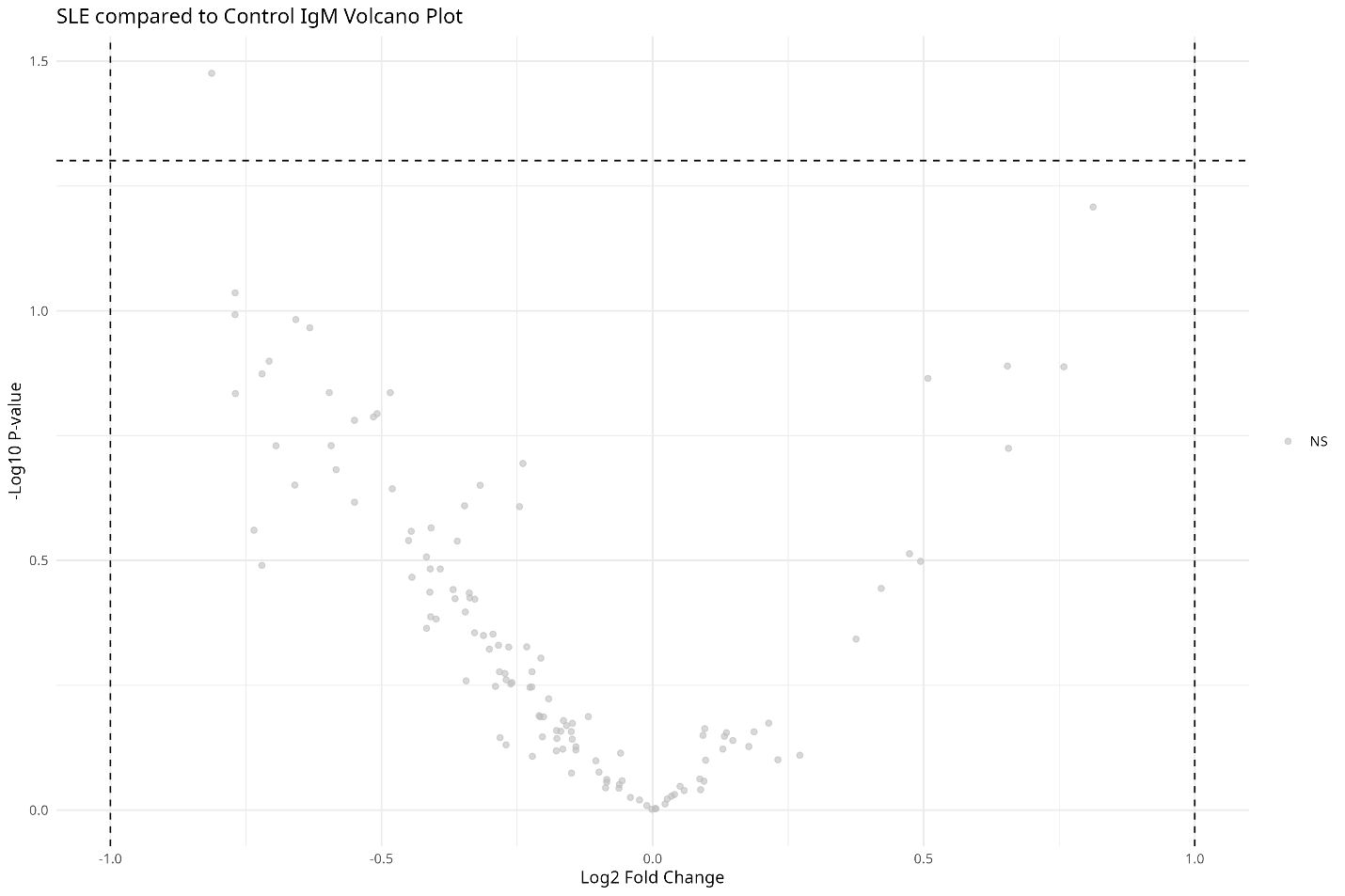

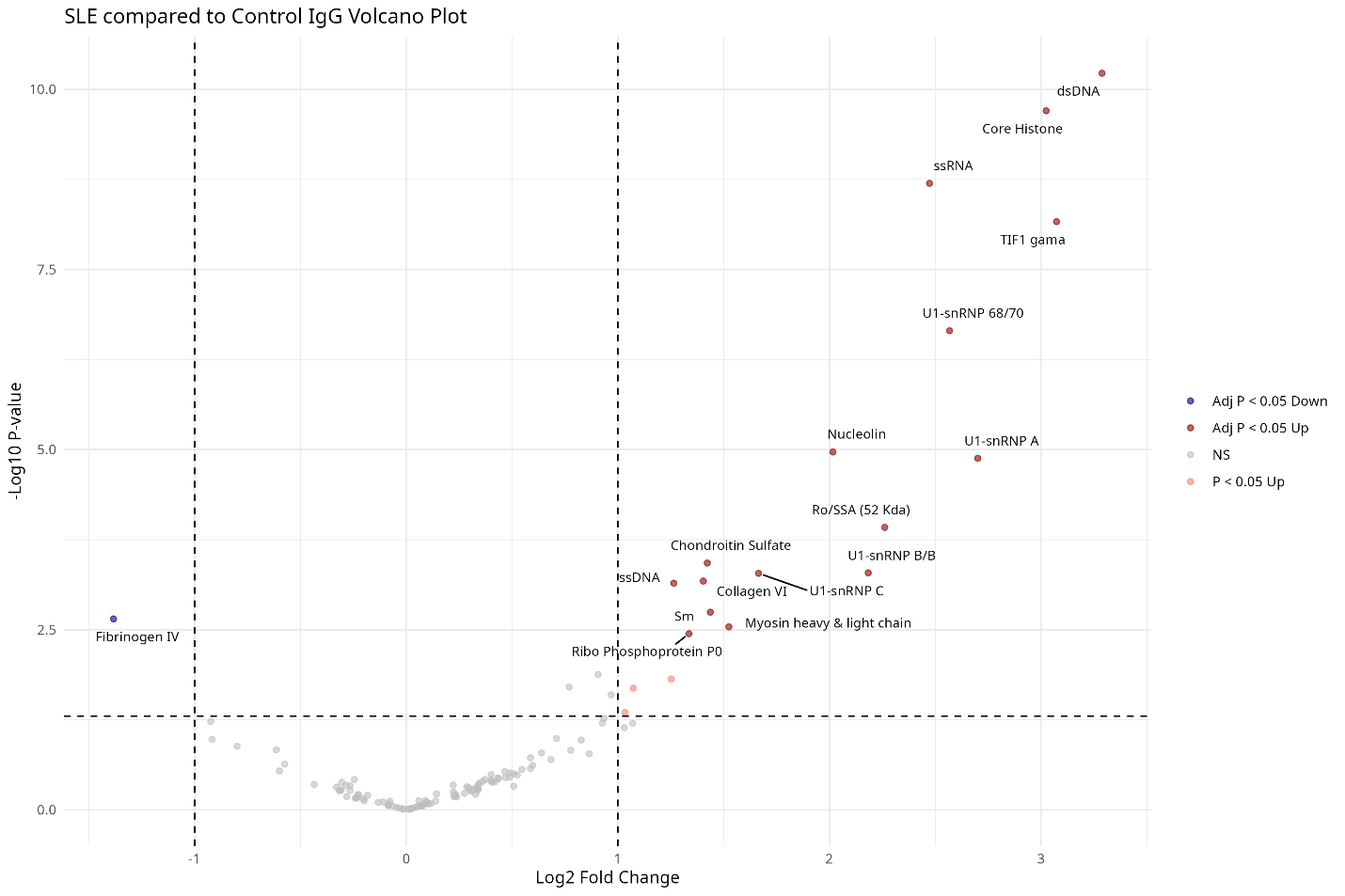
**

**Supplementary Figure 2. Differential autoantibody reactivity in systemic lupus erythematosus (SLE) compared to healthy controls.**(A) Volcano plot demonstrating differential IgM autoantibody reactivity between SLE and healthy control sera measured using the GeneCopoeia Human Autoimmune Profiling Array. The x-axis represents log2 fold change and the y-axis represents –log10 adjusted p value. Vertical dashed lines indicate fold change thresholds and the horizontal dashed line indicates the significance threshold (adjusted p < 0.05). No IgM autoantibodies remained significantly different following BH-FDR multiple comparison correction. Gray points indicate non-significant antigens. (B) Volcano plot demonstrating differential IgG autoantibody reactivity between SLE and healthy controls. Significant autoantibodies are highlighted in red and labeled where applicable, including dsDNA, core histone, ssRNA, TIF1 gamma, U1-snRNP 68/70, U1-snRNP A, Ro/SSA (52 kDa), and multiple additional nuclear antigens. Fibrinogen IV was significantly decreased in SLE compared to controls and is shown in blue. The x-axis represents log2 fold change and the y-axis represents –log10 adjusted p value. Statistical analyses were performed using limma with BH-FDR multiple comparison correction.

|  |  |
| --- | --- |

**B**

**A**

**
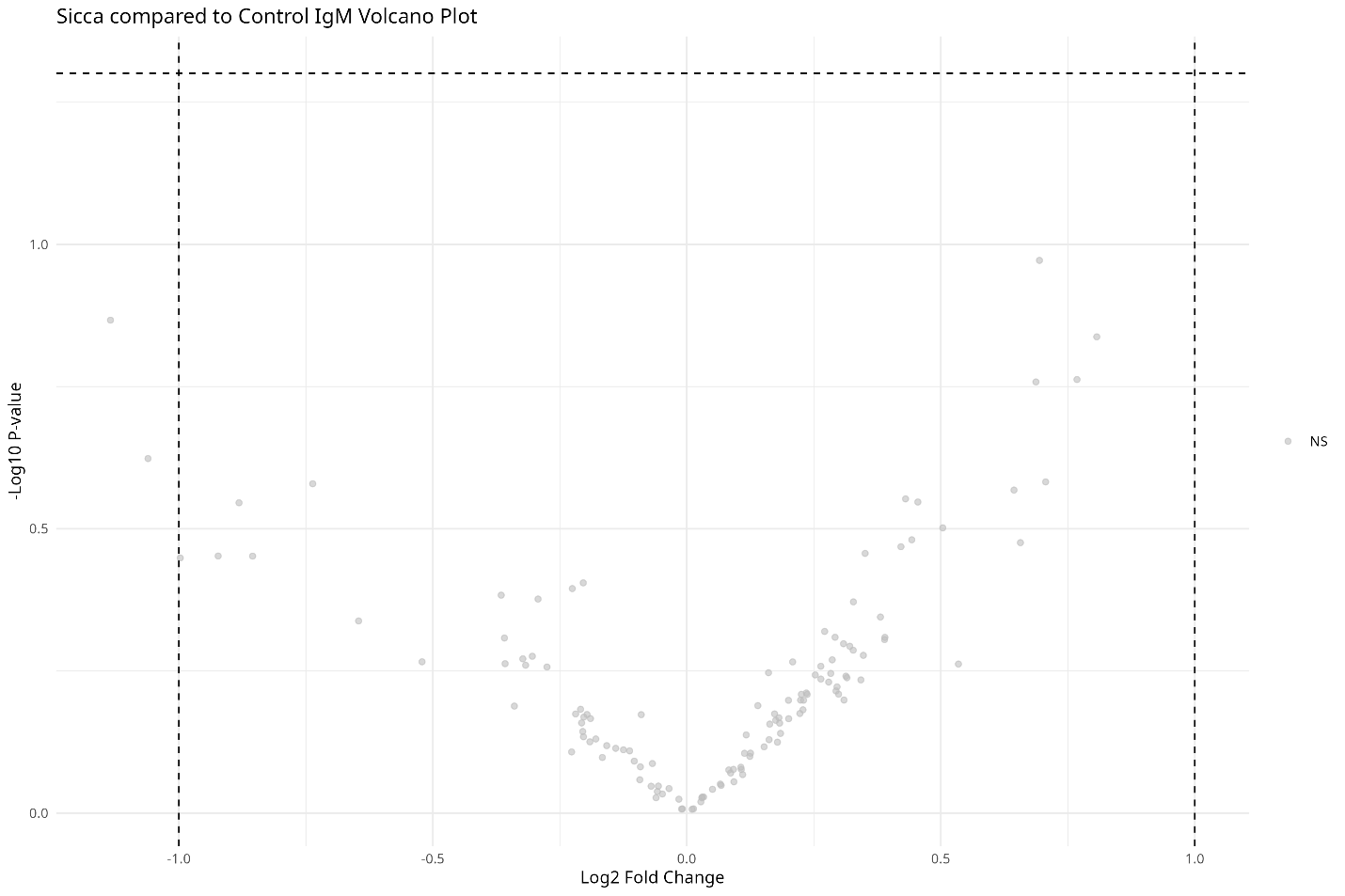

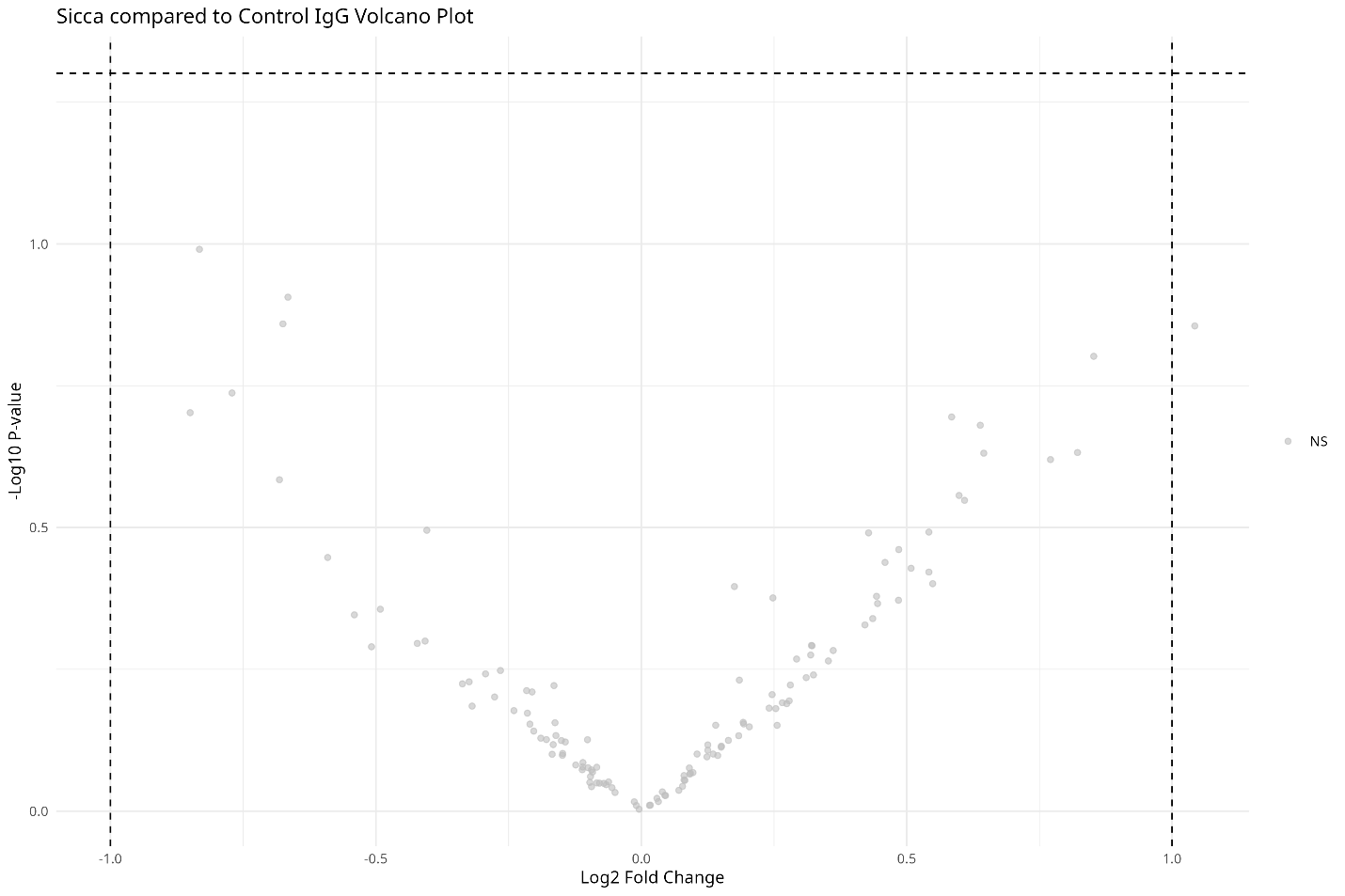
**

**Supplementary Table 3. Differential autoantibody reactivity in non-Sjögren’s Sicca (nSjD-Sicca) compared to healthy controls.**(A) Volcano plot demonstrating differential IgM autoantibody reactivity between nSjD-Sicca and healthy control sera measured using the GeneCopoeia Human Autoimmune Profiling Array. The x-axis represents log2 fold change and the y-axis represents –log10 adjusted p value. Vertical dashed lines indicate fold change thresholds and the horizontal dashed line indicates the significance threshold (adjusted p < 0.05). No autoantibodies remained statistically significant following BH-FDR multiple comparison correction. Gray points indicate non-significant autoantibodies. (B) Volcano plot demonstrating differential IgG autoantibody reactivity between nSjD-Sicca and healthy controls. The x-axis represents log2 fold change and the y-axis represents –log10 adjusted p value. Vertical dashed lines indicate fold change thresholds and the horizontal dashed line indicates the significance threshold (adjusted p < 0.05). No significantly different autoantibodies were identified following multiple comparison correction. Statistical analyses were performed using limma with BH-FDR adjustment.

|  |  |
| --- | --- |

**A**

**B**

**
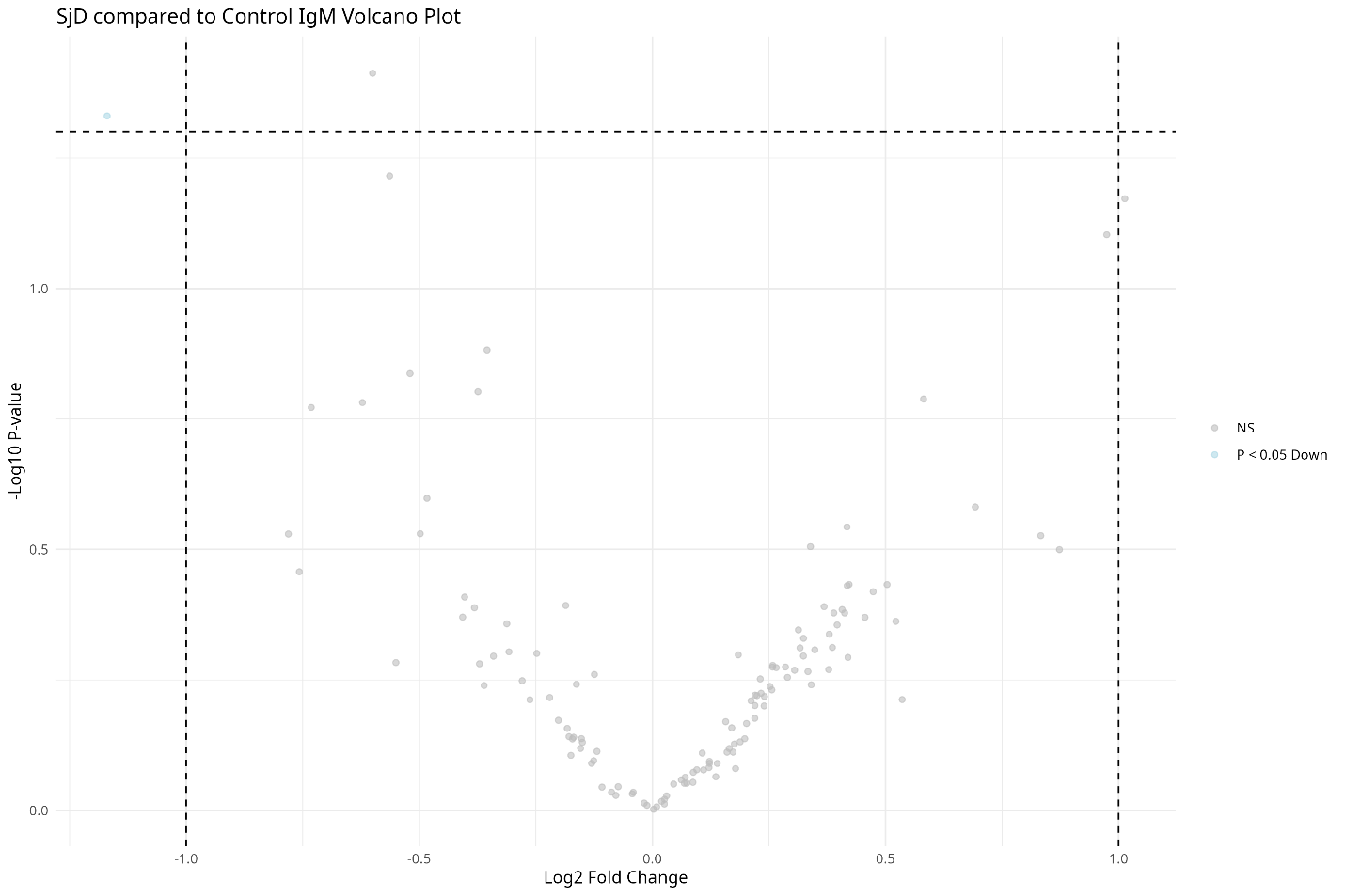
**

**Supplementary Table 4. Differential autoantibody reactivity in Sjögren’s Disease (SjD) compared to healthy controls.**(A) Volcano plot demonstrating differential IgM autoantibody reactivity between SjD and healthy control sera measured using the GeneCopoeia Human Autoimmune Profiling Array. The x-axis represents log2 fold change and the y-axis represents –log10 adjusted p value. Vertical dashed lines indicate fold change thresholds and the horizontal dashed line indicates the significance threshold (adjusted p < 0.05). No autoantibodies remained statistically significant following BH-FDR multiple comparison correction. Gray points indicate non-significant autoantibodies. (B) Volcano plot demonstrating differential IgG autoantibody reactivity between SjD and healthy controls. The x-axis represents log2 fold change and the y-axis represents –log10 adjusted p value. Vertical dashed lines indicate fold change thresholds and the horizontal dashed line indicates the significance threshold (adjusted p < 0.05). Ro/SSA (52kDa) was the only autoantibody statistically elevated in SjD compared to controls following multiple comparison correction. Statistical analyses were performed using limma with BH-FDR adjustment.

|  |  |
| --- | --- |

**B**

**A**

**
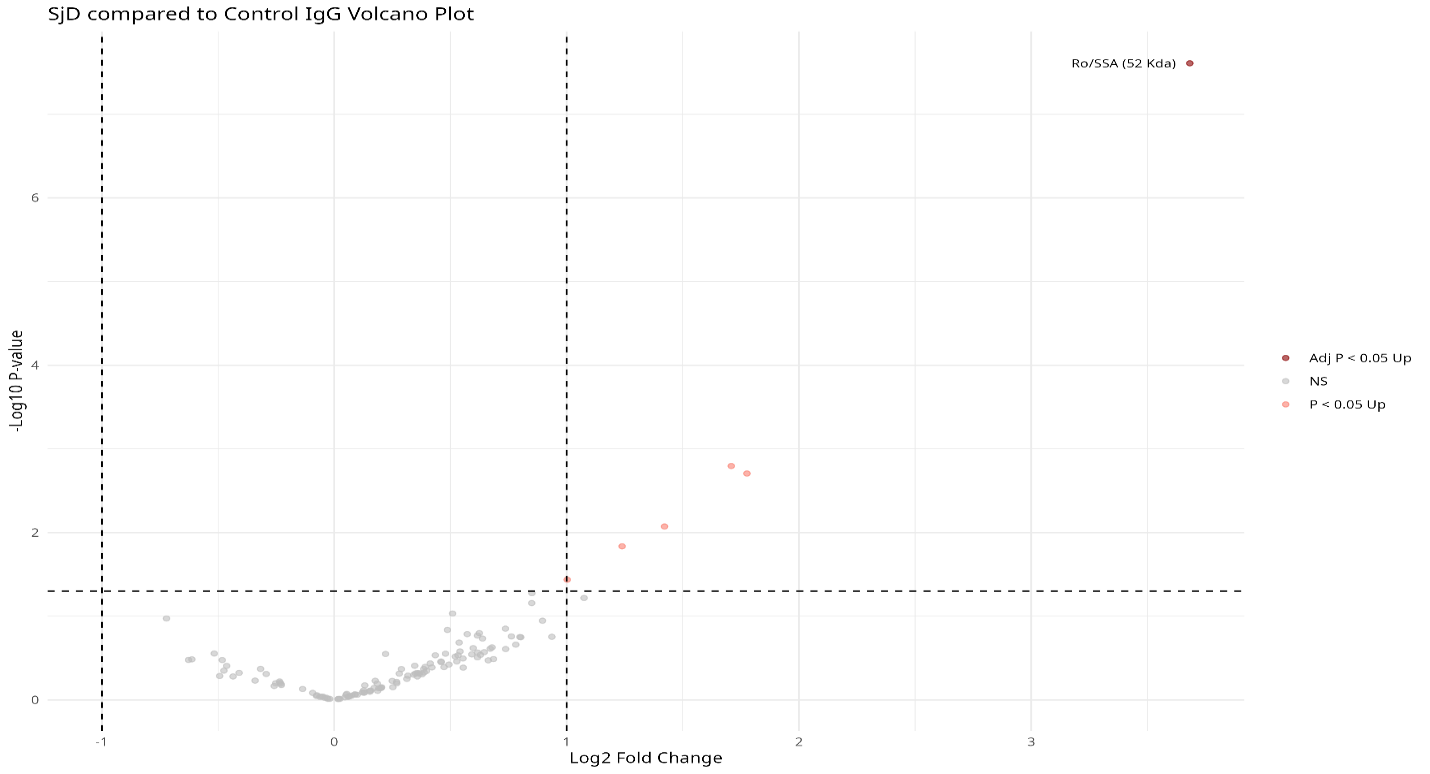
**

**Supplemental Figure 5. IgM autoantibody reactivity across clinical groups.** The heatmap is showing IgM autoantibody reactivity autoantibody score (Ab score) measuring 2 standard deviations above the control mean using the GeneCopoeia Human Autoimmune Array (120 autoantigens) for healthy controls, ILE, SLE, nSjD-Sicca, and SjD. Rows represent individual autoantibodies and columns represent individual subjects, grouped by clinical classification. Color intensity corresponds to relative autoantibody score, with darker colors indicating low signal/Ab score and warmer colors indicating higher reactivity/Ab score 2 standard deviations above the control mean.

ILE, incomplete lupus erythematosus; SLE, systemic lupus erythematosus; nSjD-Sicca, non Sjögren’s Disease – Sicca; SjD, Sjögren’s Disease.

|  |  |
| --- | --- |

**Supplemental Figure 6. Heatmap of IgG autoantibody reactivity across clinical groups.**

The heatmap is showing IgG autoantibody reactivity. Autoantibody scores (Ab scores) are calculated as 2 standard deviations above the control mean using the GeneCopoeia Human Autoimmune Array (120 autoantigens) for healthy controls, ILE, SLE, nSjD-Sicca, and SjD. Rows represent individual autoantibodies and columns represent individual subjects, grouped by clinical classification. Color intensity corresponds to relative autoantibody score, with darker colors indicating low signal/Ab score and warmer colors indicating higher reactivity/Ab Score.

ILE, incomplete lupus erythematosus; SLE, systemic lupus erythematosus; nSjD-Sicca, non Sjögren’s Disease – Sicca; SjD, Sjögren’s Disease.

|  |  |
| --- | --- |

**Supplementary Figure 7. Comparison of autoantibody positivity thresholds across disease groups (IgM).** The number of positive autoantibodies per individual was calculated using three thresholding approaches for controls (A), ILE (B), SLE (C), nSjD-Sicca (D), and SjD (E): mean + 2 standard deviations (SD), mean + 1.8 SD, and the 95th percentile. Each point represents an individual sample. Across all disease groups, less stringent thresholds (mean + 1.8 SD and 95th percentile) resulted in increased detection of positive autoantibodies compared to the mean + 2 SD approach. Statistical comparisons between thresholding methods were performed within each group using Kruskal–Wallis with Dunn’s post hoc test. ILE, incomplete lupus erythematosus; SLE, systemic lupus erythematosus; nSjD-Sicca, non Sjögren’s Disease – Sicca; SjD, Sjögren’s Disease.

|  |  |
| --- | --- |

**A**

**B**

**C**

**D**

**E**

**A**

**B**

**E**

**D**

**C**

**Supplementary Figure 8. Comparison of autoantibody positivity thresholds across disease groups (IgG).** The number of positive autoantibodies per individual was calculated using three thresholding approaches for controls (A), ILE (B), SLE (C), nSjD-Sicca (D), and SjD (E): mean + 2 standard deviations (SD), mean + 1.8 SD, and the 95th percentile. Each point represents an individual sample. Across all disease groups, less stringent thresholds (mean + 1.8 SD and 95th percentile) resulted in increased detection of positive autoantibodies compared to the mean + 2 SD approach. Statistical comparisons between thresholding methods were performed within each group using Kruskal–Wallis with Dunn’s post hoc test. ILE, incomplete lupus erythematosus; SLE, systemic lupus erythematosus; nSjD-Sicca, non Sjögren’s Disease – Sicca; SjD, Sjögren’s Disease.

|  |  |
| --- | --- |

| Comparison | Isotype | Top 5-Ranked Features Overlap Compared | Top 40-Ranked Features Overlap  Compared |
| --- | --- | --- | --- |
| ILE vs SLE | IgG | 5/5 | 21/40 |
| ILE vs SLE | IgM | 2/5 | 20/40 |

**Supplementary Table 7. Normalization sensitivity analysis of autoantibody signal processing.** Concordance was evaluated by comparing overlap among the top 5 and top 40 ranked features between preprocessing methods for both IgG and IgM datasets between ILE and SLE groups. IgG demonstrated strong concordance across normalization approaches, including complete overlap among the top 5-ranked features (5/5). IgM showed greater variability in feature ranking, although moderate overlap among the top 40-ranked features was preserved. These findings indicate that the strongest IgG signals and broader group-level autoantibody patterns remain consistent across normalization strategies despite variability among lower-ranked features.

|  |  |
| --- | --- |

**Supplementary Figure 9: Heatmaps show background-corrected, log-transformed, row normalized to z-score signal intensities for (A) IgM and (B) IgG autoantibodies across controls, ILE, SLE, nSjD-sicca, and SjD samples.** ILE, incomplete lupus erythematosus; SLE, systemic lupus erythematosus; nSjD-Sicca, non Sjögren’s Disease – Sicca; SjD, Sjögren’s Disease.

|  |  |
| --- | --- |

**B**

**A**
